# Cross-species utility of microsatellite loci for the genetic characterisation of *Anisakis berlandi* (Nematoda: Anisakidae)

**DOI:** 10.1051/parasite/2020004

**Published:** 2020-02-11

**Authors:** Eleonora Bello, Michela Paoletti, Stephen C. Webb, Giuseppe Nascetti, Simonetta Mattiucci

**Affiliations:** 1 Department of Public Health and Infectious Diseases, Section of Parasitology, Sapienza-University of Rome P.le Aldo Moro 5 00185 10 Rome Italy; 2 Department of Ecological and Biological Sciences, Tuscia University Viale dell’Università s/n 01100 Viterbo Italy; 3 Cawthron Institute 98 Halifax Street East, The Wood 7010 Nelson New Zealand

**Keywords:** *Anisakis berlandi*, Microsatellites, Genetic diversity, Nuclear markers, Sex-linkage loci

## Abstract

Eight microsatellite loci, recently developed in the species *Anisakis pegreffii,* were successfully amplified in *Anisakis berlandi*, sibling species of the *A. simplex* (s. l.) complex. They were validated on adult specimens (*n* = 46) of the parasite species, collected from two individuals of the definitive host, the long-finned pilot whale *Globicephala melas* from New Zealand waters. Among the eight loci scored, one, *Anisl 07132,* had null alleles in *A. berlandi* and was thus excluded from the subsequent genetic analysis. Two loci, *Anisl 00314* and *Anisl 10535,* were monomorphic. In addition, as also previously detected in the other species of the *A. simplex* (s. l.) complex, the *Anisl 7* locus was seen to be sex-linked, showing hemizygosity in male specimens. Differential allele frequency distributions of *A. berlandi,* with respect to those previously observed in *A. pegreffii* and *A. simplex* (s. s.), were found at some microsatellite loci. The *Anisl 7* locus provided 100% diagnosis between *A. berlandi* and *A. pegreffii,* while others resulted in 99% diagnosis between *A. berlandi* and the other two species. Simple sequence repeat (SSR) loci also allowed us to estimate the genetic differentiation of *A. berlandi* from *A. pegreffii* (*F*_st_ ≈ 0.45, Dc = 0.82) and *A. simplex* (s. s.) (*F*_st_ ≈ 0.57, Dc = 0.73). The results suggest that SSRs provide a set of candidate markers for population genetics analysis of *A. berlandi*, as well as for the investigation, through a multi-locus genotyping approach, of possible patterns of hybridisation/introgression events between *A. berlandi* and the other two *Anisakis* species in sympatric conditions.

## Abstract (fr)

Huit loci microsatellites, récemment développés chez l’espèce *Anisakis pegreffii*, ont été amplifiés avec succès chez *Anisakis berlandi*, espèce sœur du complexe *A. simplex* (s. l.). Ils ont été validés sur des spécimens adultes (*n* = 46) de l’espèce, récoltés chez deux individus de l’hôte définitif, le globicéphale commun *Globicephala melas,* des eaux néo-zélandaises. Parmi les huit loci notés, l’un, *Anisl 07132*, avait des allèles nuls chez *A. berlandi* et a donc été exclu de l’analyse génétique ultérieure. Deux loci, *Anisl 00314* et *Anisl 10535*, étaient monomorphes. De plus, comme cela a également été détecté précédemment dans les autres espèces du complexe *A. simplex* (s. l.), le locus *Anisl 7* était lié au sexe, montrant une hémizygosité chez les spécimens mâles. Chez *A. berlandi*, des distributions de fréquences d’allèles, différentielles par rapport à celles précédemment observées chez *A. pegreffii* et *A. simplex* (s. s.), ont été trouvées pour certains loci microsatellites. Le locus *Anisl 7* a fourni un diagnostic à 100 % entre *A. berlandi* et *A. pegreffii*, tandis que d’autres ont abouti à un diagnostic à 99 % entre *A. berlandi* et les deux autres espèces. Les loci des SSR ont également permis d’estimer la différenciation génétique d’*A. berlandi* par rapport à *A. pegreffii* (*F*_st_ ≈ 0,45, Dc = 0,82) et *A. simplex* (s. s.) (*F*_st_ ≈ 0,57, Dc = 0,73). Les résultats suggèrent que les répétitions de séquences simples (SSR) fournissent un ensemble de marqueurs candidats pour l’analyse génétique des populations d’*A. berlandi*, ainsi que pour l’investigation, dans une approche de génotypage multilocus, des modèles possibles d’hybridation/introgression entre *A. berlandi* et les deux autres espèces d’*Anisakis* dans des conditions sympatriques.

## Introduction

*Anisakis berlandi* Mattiucci, Cipriani, Webb, Paoletti, Marcer, Bellisario, Gibson, Nascetti, 2014 [10] is a nematode belonging to the *A. simplex* (s. l.) complex. It was first described as *A. simplex* C (see [12]), co-infecting with *A. simplex* (s. s.), at adult stages in the false killer whale *Pseudorca crassidens,* in Pacific Ocean waters (Vancouver Island) and genetically recognised, as third stage larva, in fish species from off New Zealand waters [12]. Later, a formal description was provided and *A. simplex* species C was given the nomenclatural designation *A. berlandi* [10]. Key morphological diagnostic traits between *A. berlandi* and the other species of the *A. simplex* complex (i.e. *A. pegreffii* and *A. simplex* (s. s.)) were also proposed [10]. A procrustes analysis, combining both morphological and genetic datasets on specimens belonging to the three sibling species, showed their clustering into three well-defined groups, corresponding to the three taxa [10]. Furthermore, a concatenated phylogenetic inference, combining both mitochondrial and nuclear sequences datasets, showed the existence of the three species of the *A. simplex* (s. l.) complex, as distinct phylogenetic lineages [9, 10].

Ecological data pertaining to the geographical range and host distribution revealed for *A. berlandi* a discontinuous range of distribution. This includes the Austral region: the Chilean Pacific, the South Shetland Islands, New Zealand and Australian waters, and the South African Atlantic coast [5, 9–12, 20, 21]. This species has been identified, at the adult stage, in sympatry and syntopy with *A. pegreffii,* in *Globicephala melas* and *Grampus griseus* from New Zealand, and in *G. melas* from south west Atlantic waters (South African coast) and south east Pacific waters (Chilean coast) [9]. Larval stages of *A. berlandi* have been identified in nine fish species from Austral waters off New Zealand [9], the South African coast [9], the South Shetland Islands [5], the Southern Chilean coast, and in some unusual hosts from the New Caledonian waters [20]. A few larval specimens of *A. berlandi* and *A. pegreffii* were reported in myctophids from the southern waters of the Southern Ocean (i.e. South Shetland Islands, Antarctic area) and this could be related to the introduction of these two parasites species from outside the Antarctic, through their intermediate hosts which are migrating fish species [5]. Interestingly, the very low prevalence found in *M. leonina* from the South Shetland Islands [5] could also be explained by accidental infection when the pinniped host preyed upon an infected migratory fish species [13]. Moreover, L4 stages of *A. berlandi* have also been identified in *Kogia sima* in Australian waters [21].

The mitochondrial marker mtDNA *cox2* gene locus has been found to be informative for species recognition in all the species of the genus *Anisakis* [9, 23], including the species *A. berlandi* [10]. However, among the nuclear markers, those inferred from allozymes, despite their utility in the discovery of anisakid species and their genetic identification at any life-history stage, are not a standardised approach and are not available in all laboratories for species recognition. Conversely, DNA microsatellites have recently become the alternative nuclear markers of choice to be used for species recognition and population genetic analysis of nematodes included in the *A. simplex* (s. l.) complex [8]. Some DNA microsatellite loci were developed in *A. pegreffii* and *A. simplex* (s. s.) [15]; however, no diagnostic loci between these taxa were evidenced in the study. Conversely, more recently, novel DNA microsatellite loci discovered in the species *A. pegreffii* were found to cross-amplify the species *A. simplex* (s. s.) [8]. They were also found to be of diagnostic value in the recognition of *A. pegreffii* and *A. simplex* (s. s.), and for their population genetics analysis [8].

The species *A. berlandi* has never been investigated by simple sequence repeat (SSR) markers. Thus, the aim of this study was to: (i) validate the recently developed DNA microsatellite loci on a certain number of adult specimens of *A. berlandi,* collected from metapopulations of the parasite species included in its host range and geographical distribution; (ii) evaluate the genetic diversity of *A. berlandi*, as inferred from SSR analysis; (iii) provide further diagnostic nuclear markers to be used in a multi-locus genetic approach making it possible to distinguish *A. berlandi* from the other two species of the *A. simplex* (s. l.) complex, i.e. *A. pegreffii* and *A. simplex* (s. s.), which can also be particularly useful to investigate possible hybridisation and/or introgression events between the three sibling species; and (iv) estimate genetic differentiation of *A. berlandi* with respect to these *Anisakis* spp., as inferred from the SSRs.

## Materials and methods

### DNA extraction from parasite samples

*Anisakis berlandi* samples were collected from two individuals of the definitive host species, the long-finned pilot whale *Globicephala melas* (Traill), stranded on the New Zealand coast (44°30′S–172°58′E). Nematodes collected from the stomach of their hosts were washed in saline solution and then preserved, frozen at −80 °C, as part of the collection of anisakids stored at the Department of Public Health and Infectious Diseases of Sapienza – University of Rome. Because of our previous findings of microsatellites among those developed as sex-linked loci [8], only adult nematodes were used for the genetic characterisation of the species based on SSR loci. Thus, the nematodes were first distinguished as L4-stage larvae and adults; then female and male adults were selected, according to the main morphological features that are diagnostic between sexes [10]. This was done using an optical microscope at X100–400 total magnification. A total of 46 nematodes were examined from the two individual definitive hosts. The central part of each worm’s body was then used for the molecular analysis, while the cephalic and caudal ends were stored for male and female discrimination. Out of the 46 adult specimens of *Anisakis* spp. detected, *N* = 24 females and *N* = 22 males were selected for the SSR analysis.

For the DNA extraction, a tissue portion of around 2 mg was used from each worm specimen. The cetyltrimethylammonium bromide (CTAB) extraction method was used [10]. DNA obtained was quantified using a Qubit™ dsDNA HS Assay Kit with Qubit 2.0 (Invitrogen™) [19].

### Genetic analysis for identification of *A. berlandi*

Because of the possible co-occurrence of *A. pegreffii* from the same definitive host (i.e. *Globicephala melas*) and the geographical area (i.e. New Zealand waters), as previously documented [12], the specimens (*N* = 46) of *A. berlandi* used in the present study for cross amplification of SSRs loci, were previously identified to the species level by allozyme markers and sequence analysis of the mtDNA *cox2* gene locus [10]. Standard horizontal starch gel electrophoresis was performed at the enzyme loci that have proven to be diagnostic for the species *A. berlandi* [12, 14]. Staining procedures were those previously reported [12]. For sequencing of the mtDNA *cox2* gene locus, PCR amplification was performed using the primers 211F (5′-TTT TCT AGT TAT ATA GAT TGR TTT YAT-3′) and 210R (5′-CAC CAA CTC TTA AAA TTA TC-3′) [10, 23]. PCR conditions were the same as those previously described [10].

### Cross-species amplification of microsatellite loci in *A. berlandi*

A set of eight previously identified microsatellite markers [8], named *Anisl 00185, Anisl 00314, Anisl 10535, Anisl 07132, Anisl 05784, Anisl 08059, Anisl 00875* and *Anisl 7,* were scored as potentially useful markers on the species *A. berlandi*. Our previously published primer pairs flanking these eight loci [8] were used to amplify DNA from the 46 selected individual worms. The amplification of microsatellite loci was performed by two multiplex PCR procedures: *Anisl 07132, Anisl 05784, Anisl 08059* and *Anisl 00875* by Multiplex 1; *Anisl 00185, Anisl 00314, Anisl 10535* and *Anisl 7* by Multiplex 2. Both Multiplex PCR amplifications were performed in a 10 μL reaction volume, containing 5–10 ng of genomic DNA, 5 μL of Type-it Microsatellite PCR Kit (Qiagen^®^), double distilled water, and concentrations of 10 μM labelled forward and reverse primers each. The following cycling protocol was used for the amplification for both multiplex reactions: 35 cycles with 94 °C for 30 s, 56 °C for 90 s and 72 °C for 60 s. Before the first cycle, a prolonged denaturation step (95 °C for 15 min) was included, and the last cycle was followed by a 15 min extension at 60 °C [8].

Amplified PCR products were genotyped by an external Company (Macrogen service). Individual electropherograms were analysed using GeneMapper v.4.1 software (Applied Biosystems, USA), to determine the genotype of each sample. Patterns of tri- and tetrallelic peaks in the female individuals, as possible results of tissue contamination with sperm from copulation, were not found. Genotyping errors generally associated with microsatellite analysis, such as stutter bands, the presence of null alleles and allelic drop-out were checked using MICRO-CHECKER software, version 2.2.3 [24].

### Genetic data analysis

The sequences obtained here at the mtDNA *cox2* gene were aligned using Clustal X version 2.0 software [6]. The number of alleles found at the SSR loci (A), the observed heterozygosity (*H*_*o*_), the expected heterozygosity (*H*_*e*_), the Hardy–Weinberg exact test [4], and the fixation indices (*F*_IS_, *F*_IT_ and the *F*_st_) [25] inferred from the SSRs genetic data sets were evaluated using ARLEQUIN version 3.5 software [2]. Because of the discontinuous range of distribution of the species *A. berlandi* and its genetic sub-structuring in the Pacific Ocean, as previously detected by other nuclear markers (allozymes) [12], we preferred to maintain the nematode samples collected from the two individuals of *G. melas* as separate sub-populations for the analysis of molecular variance (AMOVA). AMOVA was used to determine variance among individuals from the two definitive hosts, locus by locus, using ARLEQUIN version 3.5 [2], with 1000 permutations. Cavalli-Sforza and Edwards’s chord distance [1] and Nei’s distance values [17] were calculated from the SSR allele frequency estimates, using BIOSYS 2.0 software [22]. An unweighted pair group method with arithmetic mean (UPGMA) was generated using PHYLIP software [3], based on Nei’s [17] distance values.

## Results and discussion

### Identification of *A. berlandi* specimens

Allozyme analysis of *Anisakis* (*N* = 46 specimens) from the long-finned pilot whale corresponded to *A. berlandi*, according to alleles found at the diagnostic loci with respect to the other members of the *A. simplex* (s. l.) complex, i.e. *Pep C-1* ^*92*^, and *Mdh-1* ^*80,90*^ [14]. In addition, the sequences of 629 bp in length of the mtDNA *cox2* gene locus were obtained from the same specimens. According to the diagnostic positions, as previously described [10, 23], the (*N* = 46) specimens were assigned to the species *A. berlandi*. The sequences obtained at the mtDNA *cox2* gene (629 bp) of these specimens of *A. berlandi* matched the sequences deposited in GenBank for *A. berlandi* from our previous analysis [10]. The new sequences have been submitted to GenBank for the mtDNA *cox2* gene, and their accession numbers are as follows: MN385244, MN385245, MN385246, MN385247.

### Genetic diversity within *A. berlandi* based on microsatellite DNA loci

A total of *N* = 24 female and *N* = 22 male adult specimens of *A. berlandi* were genotyped at the eight microsatellite loci scored. Each of these markers produced unambiguous genotypes with either a single or double peak on single worms, as anticipated for single locus markers in a diploid organism. Six markers were seen to be polymorphic, with the total number of alleles varying between *A* = 2 (*Anisl 05784*) and *A* = 16 (*Anisl 7*) (Table 1). However, the remaining two loci, i.e. *Anisl 00314* and *Anisl 10535,* were monomorphic in the *A. berlandi* specimens tested here (Table 2). For the *Anisl 07132* locus, a certain number of samples repeatedly failed to amplify in *A. berlandi*, suggesting that there were null homozygotes at this locus in the parasite species. The locus exhibited an excess of homozygotes. Consistent with this, the observed heterozygotes (*H*_*o*_ *)* were significantly fewer than the expected heterozygote genotypes (*H*_*e*_), further suggesting the presence of null alleles at this microsatellite locus (Table 1). Therefore, this marker was not taken into account when other genetic data (i.e. allele frequencies, Cavalli-Sforza [1] and Nei’s [17] genetic distance values, as well as *F*_st_) were considered.

**Table 1:** Genetic diversity at six microsatellite loci in adult specimens of *A. berlandi,* analysed in the present study.

Locus		
*Anisl 00185*	*N*	46
	*H*_*o*_	0.65
	*H*_*e*_	0.85
	*p*-value	0.04
	*A*	11
*Anisl 07132*	*N*	12
	*H*_*o*_	0.25
	*H*_*e*_	0.81
	*p*-value	***
	*A*	6
*Anisl 05784*	*N*	46
	*H*_*o*_	0.04
	*H*_*e*_	0.04
	*p*-value	1.00
	*A*	2
*Anisl 08059*	*N*	46
	*H*_*o*_	0.37
	*H*_*e*_	0.31
	*p*-value	0.43
	*A*	3
*Anisl 00875*	*N*	46
	*H*_*o*_	0.09
	*H*_*e*_	0.09
	*p*-value	1.00
	*A*	3
*Anisl 7*	*N*	46
	*H*_*o*_	0.43
	*H*_*e*_	0.89
	*p*-value	***
	*A*	16

Loci *Anisl 00314* and *Anisl 10535* were monomorphic. *N* = total number of genotyped nematodes at each locus; *H*_*e*_ = expected heterozygosity; *H*_*o*_ = observed heterozygosity; *A* = number of alleles detected at each locus; *p* = indicates the significance *(p* < 0.05) value of the deviation from HWE expectation. ***p ≪ 0.001.

**Table 2:** AMOVA results for adult specimens of *A. berlandi,* collected from two individual hosts of *Globicephala melas*.

Source of variation	d.f.	Sum of squares	Variance components	% of variation	*F*-statistics
Among populations	2	0.937	−0.010	−1.61	*F*_st_ = −0.016 (n.s.)
Among individuals within populations	43	31.096	0.073	11.50	*F*_IS_ = 0.113*
Within individuals	46	26.500	0.576	90.11	*F*_IT_ = 0.100*
Total	91	58.533	0.639		

d.f. = degrees of freedom; n.s. = not significant. *p < 0.05.

**Figure 1:**
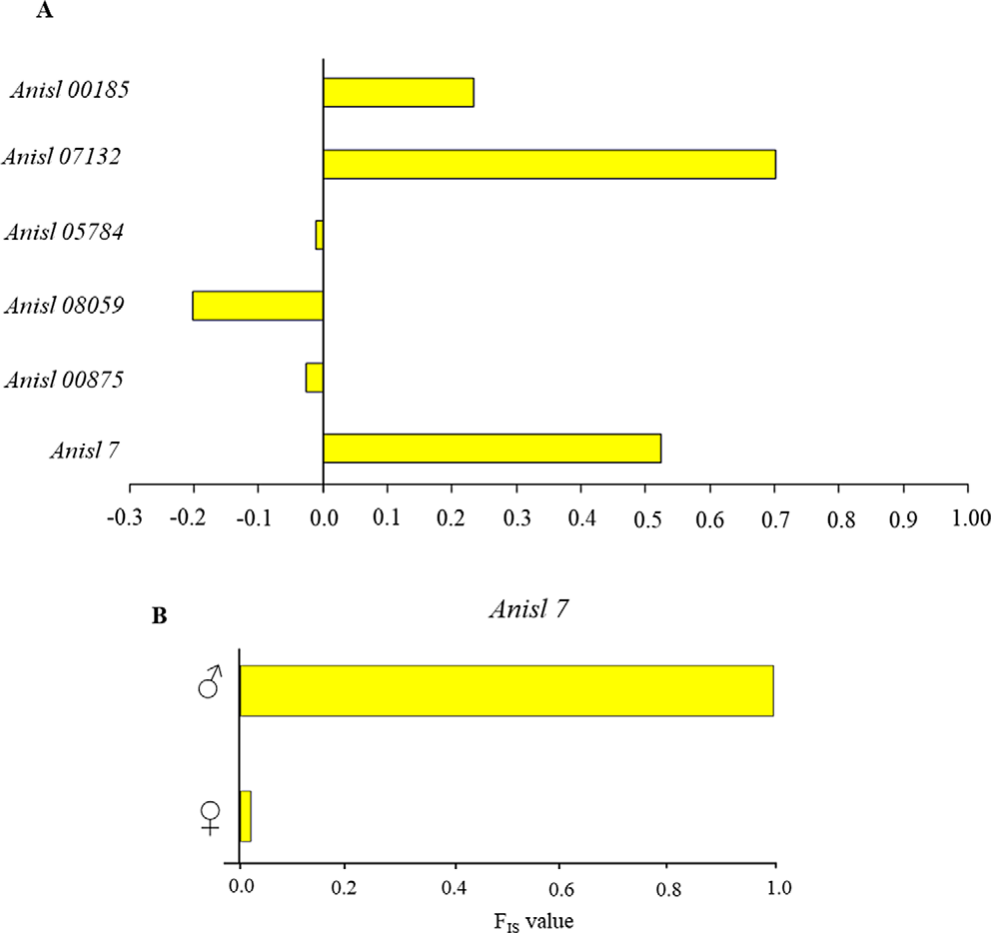
(A) *F*_IS_ calculated at the six microsatellite loci among the eight studied in *A. berlandi*. Two loci (i.e. *Anisl 00314* and *Anisl 10535*) were not included because they were found to be monomorphic. Negative values indicate heterozygous excess, while positive values indicate homozygous excess from that expected under Hardy–Weinberg Equilibrium (HWE); (B) *F*_IS_ in male and female specimens of *A. berlandi* at the sex-linked locus *Anisl 7*.

No significant departures from the Hardy–Weinberg Equilibrium (HWE) between observed (*H*_*o*_ *)* and expected (*H*_*e*_) heterozygosity were observed at the three polymorphic scored loci *Anisl 05784, Anisl 08059* and *Anisl 00875.* However, a slightly significant value (*p* = 0.04) was found at locus *Anisl 00185* (Table 1). Generally, positive values of *F*_IS_ indicated an excess of homozygote genotypes at the selected loci, while negative values indicated an excess of heterozygote genotypes from the expected HWE (Fig. 1A). Interestingly, *Anisl 7* showed statistically highly significant departures from the HWE in *A. berlandi* (Table 1), with a positive *F*_IS_ value (Fig. 1A). However, when the genotypes at *Anisl 7* were compared with those observed in adult male and female worms, it was seen that the male worms were homozygous at this locus (*F*_IS_ = 1) (Fig. 1B). Therefore, the locus *Anisl 7* appeared to be sex-linked in *A. berlandi* because of hemizygosity in the males. In fact, no significant departure (*p* = 0.13) from the HWE was observed between the observed (*H*_*o*_ = 0.88) and expected heterozygosity (*H*_*e*_ = 0.90), when considering only female worms of *A. berlandi*, scored at the *Anisl 7* locus.

After excluding the SSR locus (i.e. *Anisl 07132)* affected by null alleles and the sex-linked locus *Anisl 7*, due to hemizygosity of males, the remaining loci showed adequate genetic diversity for population-level genetic analysis. The AMOVA analysis of six nuclear markers showed that a moderate variance was significantly allocated within individuals (≈58%, with *F*_IT_ = 0.10). Furthermore, a moderately significant (*p* = 0.03) variation was found among individuals within populations (*F*_IS_ = 0.11), likely due to the locus *Anisl 00185.*

AMOVA provided no significant genetic differentiation (*F*_st_ = −0.01, *p* = 1) between the two metapopulations of *A. berlandi* considered here, as collected from the two definitive host individuals (Table 2). The last *F*_st_ value was at the same degree as those previously observed at the infra-population level within the species *A. simplex* (s. s.) and *A. pegreffii*. In fact, we found on average *F*_st_ ≈ 0.008 at the interpopulation level in *A. simplex* (s. s.) from northeast Atlantic waters. This value was *F*_st_ = 0.002 between pairs of populations geographically close to each other, such as the two samples of *A. pegreffii* from the Mediterranean Sea [8].

Additionally, based on the same common SSR loci scored in the three species of the *A. simplex* (s. l.) complex (excluding locus *Anisl 00185* found to be affected by nulls in *A. simplex* (s. s.) and locus *Anisl 00314* because of its sex-linkage in both *A. simplex* (s. s.) and *A. pegreffii* [8]), higher and significant levels of *F*_st_ were observed at the interspecific level. *A. berlandi versus A. pegreffii* resulted on average in *F*_st_ ≈ 0.45 and *F*_st_ ≈ 0.57 *versus A. simplex* (s. s.). These values are at the same degree as that previously estimated between *A. pegreffii* and *A. simplex* (s. s.) (on average *F*_st_ ≈ 0.33), based on SSR loci [8].

### Utility of microsatellite markers in *A. berlandi* identification

Allele frequencies calculated at the seven microsatellite loci (excluding *Anisl 07132*, due to the null alleles), are shown in Table 3. Most of the SSR loci studied appeared less polymorphic in *A. berlandi* in comparison with the same loci previously investigated [8] in the other two species of the complex (Table 3).

**Table 3:** Allele frequencies observed at seven microsatellite loci tested in *A berlandi,* in comparison with those we previously calculated in *A. pegreffii* and *A. simplex* (s. s) at the same SSR loci [9]. With regard to the polymorphic sex-linked locus *Anisl 7,* the most reliable estimate of allele frequencies was calculated according to the sex-linked genetic model estimate, assuming: (i) hemizygosity of males at this locus; (ii) adult female counterparts as biallelic at the sex-linked loci. The frequencies of *Anisl 00185* and *Anisl 00314* are not shown in *A. simplex* (s. s.), because these loci were affected by null alleles, as we have previously found [9].

Locus	Allele	*Anisakis berlandi*	*Anisakis pegreffii*	*Anisakis simplex* (s. s.)
*Anisl 00185*	*182*	0.01	–	–
	*185*	0.01	0.01	–
	*188*	0.01	0.04	–
	*191*	0.11	0.07	–
	*194*	0.15	0.25	–
	*197*	0.18	0.24	–
	*200*	0.28	0.15	–
	*203*	0.09	0.18	–
	*206*	0.07	0.04	–
	*209*	0.07	0.01	–
	*212*	0.02	0.01	–
*Anisl 00314*	*96*	–	0.05	–
	*100*	1.00	0.32	–
	*104*	–	0.22	–
	*108*	–	0.25	–
	*112*	–	0.13	–
	*116*	–	0.01	–
	*120*	–	0.01	–
	*124*	–	0.01	–
*Anisl 10535*	*125*	–	0.01	0.01
	*128*	1.00	0.01	0.01
	*131*	–	0.02	0.02
	*134*	–	0.10	0.91
	*137*	–	0.14	0.04
	*140*	–	0.51	0.01
	*143*	–	0.18	–
	*146*	–	0.02	–
	*149*	–	0.01	–
*Anisl 05784*	*57*	–	–	0.01
	*60*	–	–	0.01
	*63*	–	0.01	0.21
	*66*	–	0.01	0.04
	*69*	0.98	0.01	0.03
	*72*	0.02	0.01	0.01
	*75*	–	0.01	0.02
	*78*	–	0.02	0.46
	*81*	–	0.05	0.13
	*84*	–	0.07	0.05
	*87*	–	0.22	0.01
	*90*	–	0.31	0.01
	*93*	–	0.18	–
	*96*	–	0.05	0.01
	*99*	–	0.03	–
	*102*	–	0.01	–
	*105*	–	0.01	–
*Anisl 08059*	*78*	–	–	0.01
	*82*	–	0.01	0.03
	*86*	0.80	0.24	0.85
	*90*	0.19	0.03	0.05
	*94*	0.01	0.12	0.01
	*98*	–	0.13	0.02
	*102*	–	0.19	0.01
	*106*	–	0.12	0.01
	*110*	–	0.06	0.01
	*114*	–	0.04	–
	*118*	–	0.02	–
	*122*	–	0.01	–
	*126*	–	0.01	–
	*130*	–	0.01	–
	*134*	–	0.01	–
*Anisl 00875*	*142*	–	–	0.01
	*145*	–	0.01	0.01
	*148*	0.95	0.01	0.01
	*151*	0.04	0.05	0.01
	*154*	0.01	0.03	0.01
	*157*	–	0.67	0.20
	*160*	–	0.16	0.53
	*163*	–	0.04	0.16
	*166*	–	0.01	0.04
	*169*	–	–	0.01
	*172*	–	0.01	0.01
	*175*	–	0.01	–
*Anisl 7*	*216*	–	0.01	–
	*219*	–	0.22	–
	*222*	–	0.75	–
	*225*	0.02	0.02	–
	*228*	0.02	–	–
	*243*	0.02	–	–
	*252*	0.02	–	0.13
	*255*	–	–	0.48
	*258*	0.02	–	0.18
	*261*	0.07	–	0.11
	*264*	0.02	–	0.04
	*267*	0.20	–	0.03
	*270*	0.17	–	0.02
	*273*	0.11	–	–
	*276*	0.07	–	–
	*279*	0.09	–	–
	*282*	0.11	–	–
	*285*	0.02	–	0.01
	*288*	0.02	–	–
	*294*	0.02	–	–

On the contrary, in *A. berlandi,* the sex-linked locus *Anisl 7* showed several alleles. Similarly, locus *Anisl 00185* showed at least 11 distinct alleles in this *Anisakis* species (Table 3). To include the genetic data set obtained at the sex-linked locus *Anisl 7*, the most reliable estimates of allele frequencies of the parasite species were calculated only in adult specimens, according to the sex-linked genetic model estimate. This was also done considering the hemizygosity of the males and the adult female counterpart, as biallelic nematodes at the sex-linked locus (Table 3).

**Figure 2:**
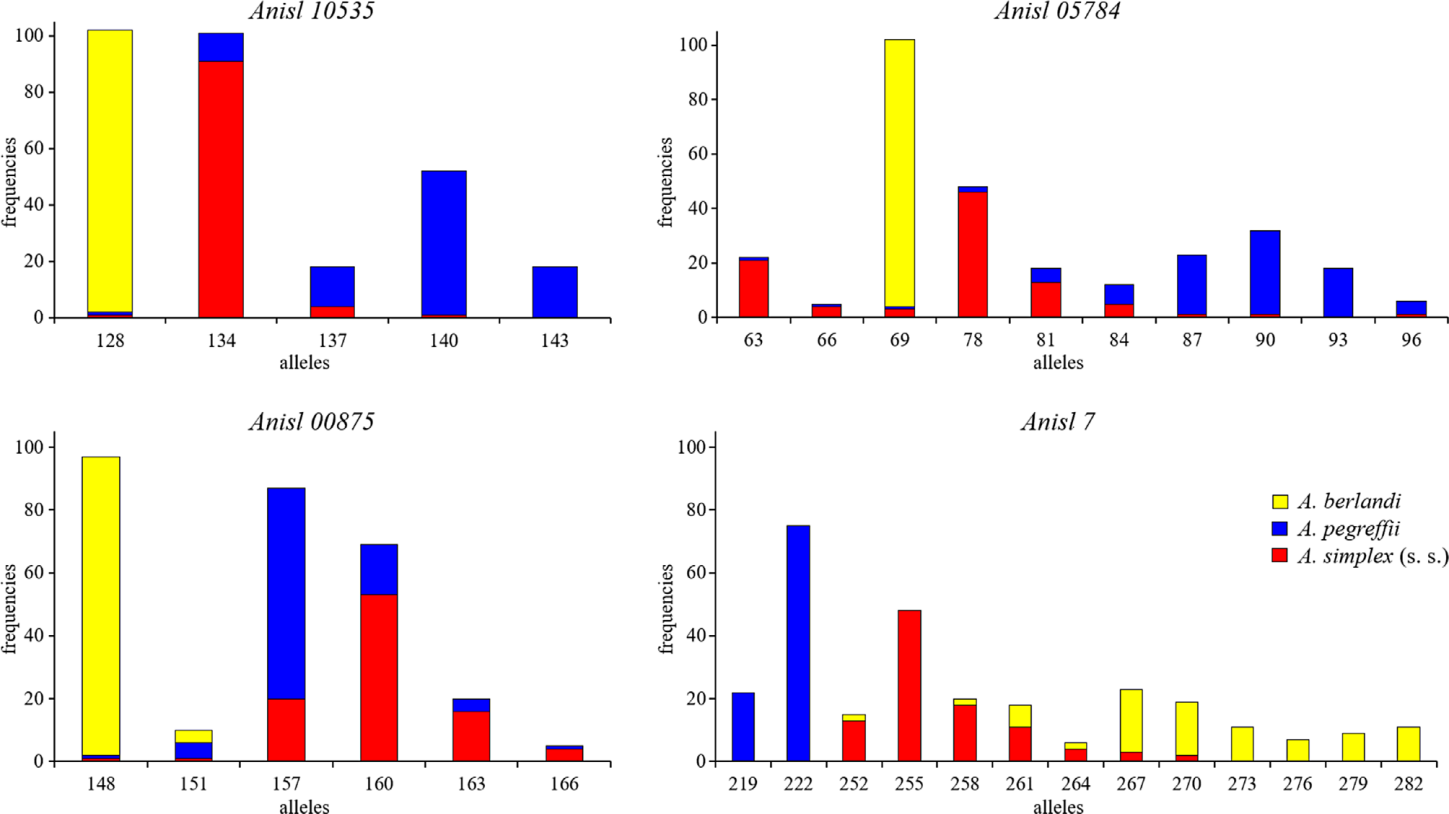
Distribution of allele frequencies of microsatellite loci in *A. berlandi* (yellow colour) shown for the partially diagnostic loci, with respect to the species *A. pegreffii* (blue colour) and *A. simplex* (s. s.) (red colour) (see Table 3). Alleles showing a frequency ≤0.03 in the three *Anisakis* spp. were not included in the graphical representation, except in cased where they occurred in common with one or in the other two *Anisakis* species at a frequency ≥0.03 (see Table 3).

It was also found that most of the amplified loci in *A. berlandi* seem to share alleles with those previously observed in *A. pegreffii* and *A. simplex* (s. s.) [8] (Table 3). However, significant differential allele frequencies in *A. berlandi* with respect to both *A. pegreffii* and *A. simplex* (s. s.) resulted in the scoring of the two SSR loci *Anisl 00875* and *Anisl 05784* (Table 3, Fig. 2). Importantly, the species *A. berlandi* showed, for instance, a high frequency (0.98%) of allele 69 at locus *Anisl 05784*, while the same allele was scored at very low frequency, i.e. 0.01% and 0.03% in *A. pegreffii* and *A. simplex* (s. s.), respectively (Table 3, Fig. 2). Similarly, at locus *Anisl 00875*, *A. berlandi* exhibited a significantly high frequency for allele *148*, which conversely occurs at very low frequency (0.01%) in both *A. pegreffii* and *A. simplex* (s. s.) (Table 3, Fig. 2). Locus *Anisl 10535* in *A. berlandi* was monomorphic for allele *128* (Table 3, Fig. 2), while the same allele was scored at a very low frequency (0.01%) in a few populations of *A. pegreffii* and *A. simplex* (s. s.) previously studied at the same locus [8] (Table 3, Fig. 2). Therefore, at this locus, the allele observed in *A. berlandi* was seen to be almost diagnostic (at 99%), allowing recognition of *A. berlandi,* with respect to *A. pegreffii* and *A. simplex* (s. s.) (Table 3, Fig. 2). The sex-linked locus *Anisl 7* in *A. berlandi* showed at least 16 distinct alleles (Table 3, Fig. 2), which are clearly distinct from two further alleles we observed in the species *A. pegreffii* [8]. Thus, the locus was 100% diagnostic between *A. berlandi* and *A. pegreffii*. Finally, locus *Anisl 00314* was monomorphic for allele *100* in the species *A. berlandi* (Table 3); this locus was found to be sex-linked in *A. pegreffii* and *A. simplex* (s. s.). Conversely, it was not possible to demonstrate sex-linkage at locus *Anisl 00314* because its monomorphic status in *A. berlandi.*

Estimates of genetic differentiation by Cavalli-Sforza & Edwards [1] chord distance, Dc, inferred from the allele frequencies calculated at those SSRs loci considered as valid (i.e. not affected by null alleles in any of the three *Anisakis* species) at the interspecific level was, on average, Dc = 0.82 and Dc = 0.73 between *A. berlandi versus A. pegreffii* and *A. simplex* (s. s.), respectively. For these estimates, allele frequencies data obtained at loci *Anisl 10535, Anisl 05784, Anisl 08059, Anisl 00875* and *Anisl 7* were included. Data from *Anisl 00314* and *Anisl 00185* were excluded from the estimation because the locus was found to be affected by nulls in *A. simplex* (s. s.), as we have previously demonstrated [8]. These values of genetic differentiation were higher than those previously reported from allozyme markers, i.e. on average, *D*_Nei_ = 0.55 and *D*_Nei_ = 0.49, between the same pairs comparison [12], even though these values were based on a larger number of loci. However, the estimates of genetic divergence are at the same scale level as that observed between the two members *A. pegreffii* and *A. simplex* (s. s.), when based on the same SSR markers: Dc = 0.62 between *A. pegreffii* and *A. simplex* (s. s.) [8].

## Conclusions

In the present study, the utility of cross-species transfer of microsatellites, previously developed in the other two closely related species of the *A. simplex* (s. l.) complex, was validated for the genotyping of *A. berlandi*. Out of the eight SSRs previously scored, only one, i.e. locus *Anisl 07132,* failed in the cross-amplification in this *Anisakis* species. Null alleles have previously been detected in other SSR loci in the species *A. simplex* (s. s.) (i.e. *Anisl 00314* and *Anisl 00185*). This is because the SSR primers were first selected in the species *A. pegreffii* [8]. The possible presence of null alleles would require careful protocol development in order to obtain consistent amplification, when cross-species amplification is tested between closely related species.

An interesting discovery in this study was that in *A. berlandi,* the SSR locus *Anisl 7* was located on the X sex chromosome, thus being sex-linked. In fact, as in the case of *A. pegreffii* and *A. simplex* (s. s.), males of *A. berlandi* are hemizygous at that locus for several alleles. This finding gives further support to the generalisation that male specimens belonging to *Anisakis* spp. are likely to possess the XO sexual karyotype, like other ascarids [16].

In spite of the low number of SSR loci so far developed in the three species of the *A. simplex* (s. l.) complex and the finding that some of them do not properly cross-amplify in all three species, the actual SSR loci to be considered as “valid” nuclear markers are of potential value in the discrimination of the three species (Table 3, Fig. 2). For instance, locus *Anisl 10535* which has shown the same alleles without significant differences in their relative proportions in *A. pegreffii* and *A. simplex* (s. s.), was found to have, instead, a single allele in *A. berlandi*, while the same allele very rarely occurred (0.01%) in the other two species (Table 3, Fig. 2). Similarly, locus *Anisl 7,* which was diagnostic at 100% between *A. pegreffii* and *A. simplex* (s. s.) [8], also had full diagnostic value between *A. pegreffii* and *A. berlandi*. In the present genetic analysis, no individuals showing evidence of mixed ancestry genotypes were detected between *A. berlandi* and *A. pegreffii*, despite the collection of *A. berlandi* specimens from a geographical area where sympatry between *A. pegreffii* and *A. berlandi* could occur [9].

**Figure 3:**
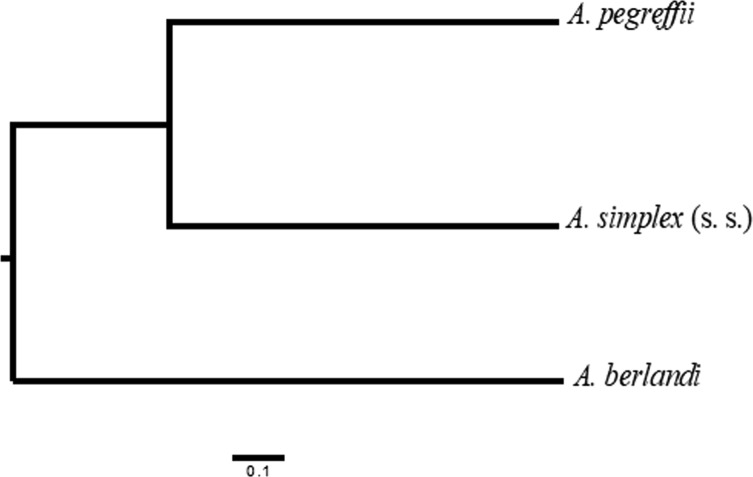
Unweighted pair group method of analysis (UPGMA) cluster based on Nei’s genetic distance values, inferred from allelic frequencies calculated at five microsatellite loci (i.e. *Anisl 10535, Anisl 05784, Anisl 00875, Anisl 08059* and *Anisl 7*), showing the genetic relationship between *A. berlandi* and the other two members of the *A. simplex* (s. l.) complex (based on the allele frequencies found, on average, at the same loci in *A. pegreffii* and *A. simplex* (s. s.) in our previous analysis [8].

The SSR nuclear markers studied here also showed the clear distinction of *A. berlandi* from the other two taxa of the same complex, as inferred from the *F*_st_ and Dc genetic differentiation values (Fig. 3). Interestingly, the topology of the clustering analysis (Fig. 3), obtained here by UPGMA, appears similar to that observed and inferred from our previous SSR studies [8], as well as from mitochondrial and nuclear markers [10, 23]. The clear distinctiveness of the taxon *A. berlandi*, as stated above, also highlights the utility of the detected SSRs in the identification of this species, since SSRs achieve a high discriminatory power in a nuclear multilocus genotyping approach.

The validated SSR loci in the present work and in our previous studies [8] will be applicable for future investigation of population genetic structure, at the intraspecific level, in *A. berlandi* collected from intermediate/paratenic and definitive hosts of other oceanographic waters, where the species also occur. Because of the discontinuous range of *A. berlandi* including Pacific Canada and Austral Regions [9], possible data acquired in future analysis, based on SSRs scoring, and gene flow estimation, would add knowledge about the genetic sub-structuring of this parasite species, as we previously detected by allozyme markers [12]. To this end, allozyme analysis, despite its powerful role in population genetics studies and species detection of anisakids, has the disadvantage of not being used as a standardised method. In contrast SSRs have been found to be a suitable and standardised nuclear tool to investigate the genetic variability and population genetic structure of the other two members of the *A. simplex* (s. l.) complex, i.e. *A. pegreffii* and *A. simplex* (s. s.). [8].

In addition, future scoring of the SSRs loci in other larval and adult populations of *A. berlandi* would clarify whether the *F*_IT_ value higher than zero observed here in *A. berlandi*, as we have previously found in both *A. pegreffii* and *A. simplex* (s. s.) [8], indicates a certain subdivision between subpopulations of the parasite species, hosted by different definitive and intermediate/paratenic fish hosts. However, the high polymorphism observed at the scored SSR loci requires a larger number of specimens to be studied in the parasite populations collected from different definitive and intermediate/paratenic host species, in order to find causes for these differences at the infrapopulation level.

Finally, in a multi-nuclear genotyping approach, SSR markers provide a powerful means to investigate, including also SNP polymorphisms detected in other nuclear genes [7, 18], the detection of possible patterns of hybridisation/introgression events between the three species of the *A. simplex* (s. l.) complex, in sympatric areas and syntopic conditions [9].
